# Increased Responsiveness of Peripheral Blood Mononuclear Cells to In Vitro TLR 2, 4 and 7 Ligand Stimulation in Chronic Pain Patients

**DOI:** 10.1371/journal.pone.0044232

**Published:** 2012-08-28

**Authors:** Yuen H. Kwok, Mark R. Hutchinson, Melanie G. Gentgall, Paul E. Rolan

**Affiliations:** 1 Discipline of Pharmacology, School of Medical Sciences, University of Adelaide, Adelaide, South Australia, Australia; 2 Discipline of Physiology, School of Medical Sciences, University of Adelaide, Adelaide, South Australia, Australia; 3 Pain and Anaesthesia Research Clinic, University of Adelaide, Adelaide, South Australia, Australia; 4 Pain Management Unit, Royal Adelaide Hospital, Adelaide, South Australia, Australia; Johannes Gutenberg University of Mainz, Germany

## Abstract

Glial activation via Toll-like receptor (TLR) signaling has been shown in animals to play an important role in the initiation and establishment of chronic pain. However, our ability to assess this central immune reactivity in clinical pain populations is currently lacking. Peripheral blood mononuclear cells (PBMCs) are an accessible source of TLR expressing cells that may mirror similarities in TLR responsiveness of the central nervous system. The aim of this study was to characterize the IL-1β response to various TLR agonists in isolated PBMCs from chronic pain sufferers (on and not on opioids) and pain-free controls. Venous blood was collected from 11 chronic pain sufferers on opioids (≥ 20 mg of morphine / day), 8 chronic pain sufferers not on opioids and 11 pain-free controls. PBMCs were isolated and stimulated *in vitro* with a TLR2 (Pam3CSK4), TLR4 (LPS) or TLR7 (imiquimod) agonist. IL-1β released into the supernatant was measured with ELISA. Significantly increased IL-1β expression was found in PBMCs from chronic pain sufferers (on and not on opioids) compared with pain-free controls for TLR2 (*F*_(6, 277)_ = 15, *P*<0.0001), TLR4 (*F*_(8, 263)_ = 3, *P* = 0.002) and TLR7 (*F*_(2,201)_ = 5, *P* = 0.005) agonists. These data demonstrate that PBMCs from chronic pain sufferers were more responsive to TLR agonists compared with controls, suggesting peripheral cells may have the potential to become a source of biomarkers for chronic pain.

## Introduction

Traditionally, the mechanisms underlying chronic pain have thought to be solely neuronally based. However, there is now overwhelming evidence from animal studies [1], [2], [3], [4], [5], [6], [7], [8] that pro-inflammatory activation of the immune-like cells (glia), found in the central nervous system (CNS), are central to the initiation and the progression of chronic pain such as neuropathic pain [9], [10]. Despite this however, there remains a lack of human data demonstrating that these pathways are relevant to clinical pain.

Toll-like receptors (TLRs) are a key detection system via which glia become activated and release pro-inflammatory cytokines [8]. TLRs form a key part of the innate immune system, capable of the detection of “self” molecules known as danger-associated molecular patterns (DAMPs) or “non-self” molecules produced by microorganisms known as pathogen-associated molecular patterns (PAMPs). Examples of exogenous TLR agonists include: lipopeptides recognized by TLR2 homodimer or by TLR2-TLR1 or TLR2-TLR6 heterodimers, lipopolysaccharide by TLR4 and single-stranded viral RNA by TLR 7 [11]. Once TLRs bind PAMPs, a series of signal transduction cascades occur, leading to the activation of NF-κB and c-Jun N-terminal kinases (JNKs) [12] which induce the transcription of genes encoding cytokines and chemokines that are involved in the initiation of the inflammatory process [13].

TLRs have been implicated in preclinical models of chronic pain [8]. Blocking of TLRs, either genetically [14], [15], or pharmacologically [16], has been found to reduce microglial activation, reduce pro-inflammatory cytokine level and produce a reduction in experimentally-induced neuropathic pain in animals [8]. Therefore, the propensity of an individual towards proinflammatory TLR signaling could be a critical contributor to chronic pain states.

Opioids are frequently prescribed for moderate to severe pain but their efficacy is limited by adverse effects such as tolerance [17]. Interestingly, it has recently been demonstrated that opioids such as morphine, are also TLR4 agonists [18], [19], [20], [21] therefore it can induce central immune signaling events that reduces opioid analgesia and contribute to hyperalgesia [19], [22]. The impact of opioid exposure on TLR signaling in clinical pain populations is highly relevant and needs to be examined in this study.

Despite the clear evidence from *in vitro* and *in vivo* preclinical models that glial activation and TLR signaling play a critical role in chronic pain and opioid responses, determining if the same occurs in human remains difficult due to the general inaccessibility of the CNS compartment. Excitingly, given the functional similarities between TLR signaling of immune cells in the periphery and in the CNS, we hypothesize that indirect evidence of activity of the TLR pathways in the CNS may be obtained by examining the activity of the TLR pathways in the peripheral immune system. Therefore, the TLR responsivity of peripheral immune cells from different patient populations where TLR activity is hypothesized to contribute to the disease state should be different when compared to healthy controls. Such information could lead to improve mechanistic understandings of pain and to the discovery of objective pain biomarkers.

Thus, in this study we have examined *in vitro* responsiveness to 3 different specific TLR agonists in peripheral blood mononuclear cells from chronic pain patients maintained on opioids, opioid-free chronic pain patients and opioid-free healthy controls, to determine whether chronic pain patients have increased responsiveness compared to pain-free controls. Moreover, we sought to investigate the relationship between response scores obtained from the cold pain test with IL-1β expression from PBMCs after TLR agonist stimulation. We hypothesized that chronic pain patients would show increased responsiveness to TLR agonists and that patients on opioids would show a further increase in TLR-mediated IL-1β response.

## Materials and Methods

### Study Participants

This study was conducted at the Pain and Anaesthesia Research Clinic, Royal Adelaide Hospital, Adelaide, Australia. Ethical approval was obtained from the Human Research Ethics Committee of the Royal Adelaide Hospital, Adelaide, South Australia.

All participants gave written informed consent to participate after a detailed oral explanation of the study. All participants were paid $50 for their inconvenience upon completion of the study. Chronic pain patients were recruited from the general public through advertisements and from a pain management unit. Healthy participants were recruited from the Pain and Anaesthesia Research Clinic's healthy participant database. Thirty participants were recruited to the study and assigned into three groups according to their pain and opioid use status. Groups 1 and 2 consisted of chronic pain sufferers. Participants had to experience pain at least five days a week and for at least 3 months. There was no minimum pain score. For Group 1, patients had to be taking ongoing opioid therapy at a dose equivalent to at least 20 mg of oral morphine per day (calculated according to a comparative opioid chart [23]). Group 2 patients were not receiving chronic opioid therapy. Group 3 was the control group in which participants had no clinically significant chronic pain and were not taking opioids or other analgesics.

For all participants the key inclusion criteria were the following: aged between 18 and 65 years, be in good general health (other than chronic pain for Groups 1 and 2) without clinically significant renal, hepatic, cardiac or other diseases. Key exclusion criteria were: use of any immunosuppressant drugs (e.g. azathioprine); presence of an active inflammatory process; a clinically significant infection in the previous 4 weeks; Raynaud's phenomenon or disease or any other condition associated with abnormal sensitivity to cold pain; a positive urine screen for illicit drugs (except for prescribed opioids), pregnancy and/or lactation, and have a known history of hepatitis B, C or HIV.

### Design

The study was of cross sectional design. The study physician was aware of the pain and medication status of the participants, but personnel performing the cold pain tests and laboratory evaluations of immune cell reactivity were blinded to the pain and opioid status of the participant.

### Study schedule

#### Visit 1

On the first visit, information on pain history and medication use was recorded. Vital signs were recorded and participants underwent the cold pain test as previously described [24] in which cold pain threshold and endurance were assessed. The temperature of the room was set at 22.5°C at all times. Briefly, the cold pain test consisted of two temperature-controlled water baths of 34.5–35.5°C and 0.5–1.5°C. A water pump was placed in the cold-water container to prevent laminar warming around the immersed limb. Each participant's non-dominant forearm and hand (fingers wide apart) was placed vertically into the warm water for exactly 2 min so that all participants' starting temperature was the same. At 1 min 45 s, a blood pressure cuff was inflated to a pressure 20 mmHg below the diastolic blood pressure (to minimize the role of blood flow in determining the reaction to cold). At exactly 2 min, the forearm was placed into the cold water bath. The participant's eyes were covered for the entire procedure to minimize distraction. Once the arm was immersed in the cold water bath, participants were asked to indicate when they first experience pain (cold pain threshold), then asked to leave their arm submerged until they could no longer endure the pain (cold pain endurance) with a maximum cut-off time of 3 min. Upon removal of the arm from the cold water bath, participants were asked to indicate when the pain ceased. Endpoints were measured as time (s). Participants performed the cold pain test twice with a 20 min interval and the average was recorded.

#### Visit 2

On visit 2, information on health status, pain severity and pain medications were checked and recorded. Five ml of blood was collected into tubes containing EDTA and was sent for standard haematologic profile. Twenty-seven ml of blood were collected into tubes containing EDTA and left at room temperature for 1 h until isolation of peripheral blood mononuclear cells (PBMCs). PBMCs were isolated using Optiprep (Axis-Shield PoC AS, Oslo, Norway) as directed by the manufacturer using the mixer flotation method. Control wells minus the TLR agonist were also included. Isolated cells were diluted to 1×10^6^ cells·ml^−1^ in enriched RPMI 1640 (10% foetal calf serum and 1% penicillin) and plated into 96 well plates (Nunc, Roskilde, Denmark) (100 ml per well). After allowing the PBMCs to sit for an hour, a range of concentrations of agonists were added into the wells in triplicate. TLR2 agonist: synthetic triacylated lipoprotein: Pam3CSK4 from 13 pg·ml^−1^ to 1 µg·ml^−1^ (Sigma); TLR4 agonist: lipopolysaccharide: LPS from 6 pg·ml^−1^ to 10 µg·ml^−1^ (Sigma); and TLR7 agonist: imiquimod from 50 pg·ml^−1^ to 100 µg·ml^−1^ (Sigma). Plates were incubated for 20 h at 37°C, 5% CO_2_ in a humidified environment (Thermoline Scientific, Australia).

For non-TLR stimulation of inflammatory pathways, monosodium urate (MSU) crystals (suspended in phosphate buffered saline) (Sigma) primed with a low concentration of LPS (13 pg/mL) were used to stimulate the isolated PBMCs. The concentration of MSU crystals used ranged from 50 pg·ml^−1^ to 10 µg·ml^−1^. MSU crystals are known activators of NALP3 or cryopyrin [25]. NALP3 consists of a protein complex that regulates the activity of caspase-1 which then cleaves pro IL-1β to IL-1β. However to respond to MSU crystals PBMCs require priming with pro-IL-1β hence we used a low concentration of LPS (TLR4 ligand) (13 pg·ml^−1^). Our data show that the concentration selected for LPS alone cause minimal release of IL-1β.

### IL-1β assay

IL-1β levels were determined by a commercially available ELISA (IL-1β ELISA; BD Bioscience, Australia) according to the manufacturer's instructions. The absorbance was quantified on a BMG Polarstar microplate reader (BMG Labtechnologies, Offenburg, Germany) at 450 nm with absorbance at 570 nm subtracted as per manufacturer's instructions. The manufacturer's limit of quantification of 0.8 pg·ml^−1^ was used.

### Statistical analysis

Graphpad Prism 5.0 (GraphPad Software; San Diego, CA) was used for all statistical analysis and fitting of concentration-response curves. The Kruskal-Wallis test was used to assess the cold pain endurance scores. Student's t-test was used to calculate the difference between duration of chronic pain. The haematology data was normalized by log transformation and analyzed with one-way ANOVA. Age and the comparison between IL-1β expressions with the three groups at the unstimulated level were analyzed using one-way ANOVA. The concentration curves for TLR2, TLR7 agonists and MSU crystals were assessed using a sigmoidal concentration response equation. Assessments were first conducted to determine the type of model that best described the data using *F*-tests. In this case either 3 or 4 parameter sigmoidal concentration response curves were tested. In all cases except the biphasic TLR4 response described below a 3 parameter model was found to describe the data the best. The bottom responses were fixed at a value of 0 but other parameters were allowed to vary. For TLR4 a modified biphasic sigmoidal equation was used (Y = (YMin + (YMax-YMin)/(1+10?((LogEC50-X)*HillSlope)))+(YMin2 + (YMax2-YMin2)/(1+10?((LogEC502-X)*HillSlope2)))). The *F*-tests were used to ask the question of whether the best fit curves with the selected parameters (Emax, Emin and EC_50_) differ so that group differences could be identify in the IL-1β expressed by PBMCs post TLR agonist/ MSU crystals stimulation. All significance was set at *P*<0.05.

## Results

### Demographic data

Basic demographics are listed in Table 1. Eleven chronic pain sufferers on opioids (referred to as CP+O group) (7 female, 4 male, (min-max) 33–65 years old; mean age 53), eight chronic pain sufferers not on opioids (referred to as CP group)(6 female, 2 male, 36–65 years old; mean age 52) and eleven pain-free participants (7 female, 4 male; 36–61 years old; mean age 51) took part in the study. Additional information of all chronic pain participants can be found in Table S1. The average duration of pain in the CP+O group was 10 years (min-max; 2–28) and for CP group was 4 years (1–10) (*P* = 0.08). The mean daily dose (oral morphine equivalent) taken by CP+O was (mean ± SEM) 85±15 mg and 64% were also on other pain modifying medications such as pregabalin and amitriptyline. Thirty-eight percent of the CP group was on oral analgesics such as ibuprofen and gabapentin and 50% only took oral analgesics when required.

**Table 1:** Demographic summary.

	Chronic pain + Opioids	Chronic pain – Opioids	Control	*P*
**Gender (M/F)**	4/7	2/6	4/7	-
**Age (Years)**	53±3	52±4	51±3	0.89
**Oral morphine equivalent dose (per day) (mg)**	85±15	None	-	-
**Duration of chronic pain (Years)**	10±3	4±1	None	0.08

Data were collected from medical and family history. Data are expressed as mean ± S.E.M. One-way ANOVA and Student's t-test was used to determine significant differences (P-values shown).

### Cold pain endurance score

The cold pain endurance score obtained from the average of two testing sessions for CP+O group was: (median (interquartile range)) 28 (24–144) s, CP group was 32 (17–105) s and pain-free was 47 (28–106) s. There were no significant differences between either chronic pain group compared with pain-free participants (NS; *P* = 0.8).

### Haematology

There were no differences in absolute or relative white cell subtype counts (total white cell count, neutrophils, lymphocytes, monocytes, eosinophils and basophils) between the groups.

### In vitro stimulation with TLR agonists

The basal level (unstimulated) of IL-1β expression was the same for all three groups: (Mean ± SEM) CP+O group: 1±0.2 pg·ml^−1^, CP group: 0.9±0.2 pg·ml^−1^ and for pain-free controls: 1±0.2 pg·ml^−1^ (NS, *P* = 0.9).

#### (A) TLR2 agonist: Pam3CSK4

**Figure 1:**
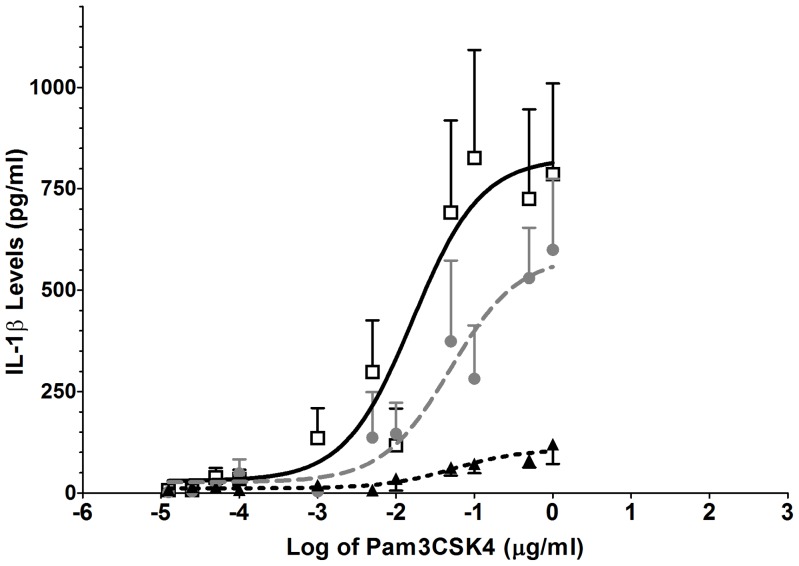
TLR 2 agonist stimulation caused significant enhanced release of IL-1β in chronic pain patients. Isolated white cells obtained from chronic pain sufferers on opioids (□), chronic pain sufferers not on opioids (•) and pain-free controls (▴) were stimulated with a range of Pam3CSK4 (TLR2) concentrations (13 pg·ml^−1^ to 1 µg·ml^−1^) to generate the response curves and resulted in significant group differences (P<0.0001). Error bars on graphs represent standard error of the mean.

The observed concentration-response curves were mono-phasic, with the pain-free group appearing almost non-responsive to the TLR2 agonist Pam3CSK4. However, Pam3CSK4 induced significant concentration-dependent increases in IL-1β release in both pain groups with the CP+O group being the most responsive. The clear separation between the three groups resulted in an overall significant group effect in response to Pam3CSK4 (*F*_6, 277_ = 15, *P*<0.0001; see Figure 1). These differences were also reflected in significantly different EC_50_ values: CP+O, 16 ng·ml^−1^ (95% confidence interval: 4 to 69 ng·ml^−1^); CP, 49 ng·ml^−1^ (95% CI: 12 to 210 ng·ml^−1^); and PF, 53 ng ml^−1^ (95% CI: 10 to 282 ng·ml^−1^). The E_max_ estimates were also significantly different with CP+O 827 pg·ml^−1^ (95% CI: 622 to 1031 pg·ml^−1^); CP, 584 pg·ml^−1^ (95% CI: 401 to 766 pg·ml^−1^); and PF 108 pg ml^−1^ (95% CI: 71 to 144 pg·ml^−1^).

#### (B) TLR4 agonist: LPS

**Figure 2:**
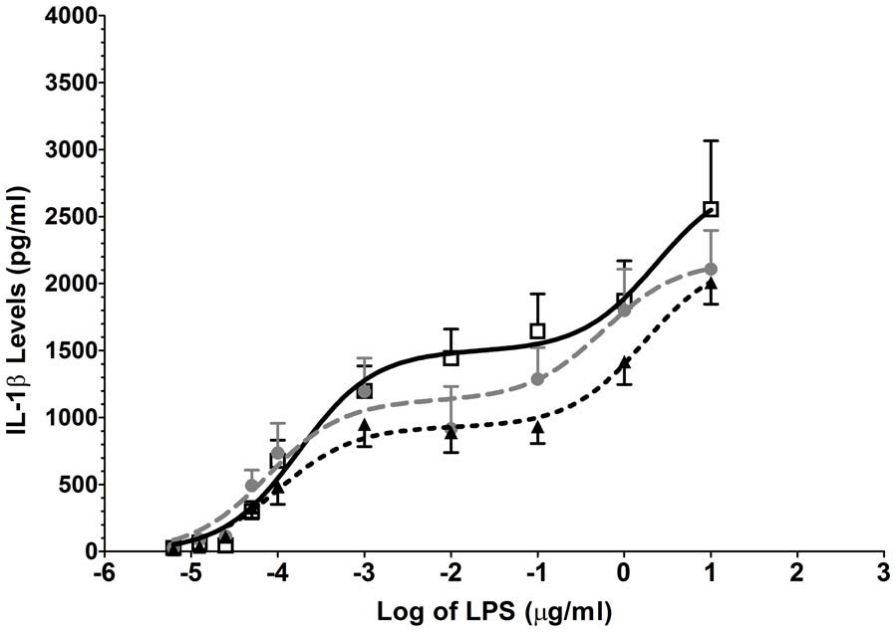
TLR 4 agonist stimulation caused significant enhanced release of IL-1β in chronic pain patients. Isolated white cells obtained from chronic pain sufferers on opioids (□), chronic pain sufferers not on opioids (•) and pain-free controls (▴) were stimulated with a range of LPS (TLR4) concentrations (6 pg·ml^−1^ to 10 µg·ml^−1^) to generate the response curves and resulted in significant differences (P = 0.002). Error bars on graphs represent standard error of the mean.

The TLR4 agonist LPS induced significant concentration-dependent increases in IL-1β release in all groups. The observed concentration-response curve was best fitted to a biphasic curve, suggesting both low and high affinity systems, combining to produce the overall concentration curve. An increased response was observed for both chronic pain populations when compared with the controls with the CP+O group being the most responsive. This resulted in a significant group difference in the IL-1β release concentration-response (*F*_8, 263_ = 3, *P* = 0.002; see Figure 2). The EC_50_ of CP+O was 16 ng·ml^−1^ (95% CI: 4 to 69 ng·ml^−1^); CP, 49 ng·ml^−1^ (95% CI: 12 to 210 ng·ml^−1^); PF 53 ng·ml^−1^ (95% CI: 10 to 282 ng·ml^−1^). The Emax were 827 pg·ml^−1^ (95% CI: 622 to 1031 pg·ml^−1^) for CP+O, 584 pg·ml^−1^ (95% CI: 401 to 766 pg·ml^−1^) for CP and 108 pg·ml^−1^ (95% CI: 71 to 144 pg·ml^−1^) for PF.

#### (C) TLR7 agonist: Imiquimod

**Figure 3:**
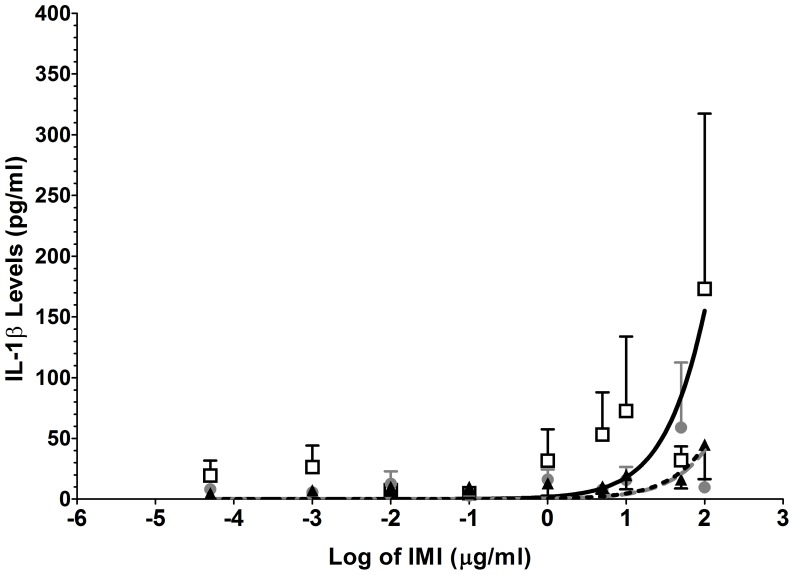
TLR 7 agonist stimulation allowed the differentiation between chronic pain patients and pain-free participants. Isolated white cells obtained from chronic pain sufferers on opioids (□), chronic pain sufferers not on opioids (•) and pain-free controls (▴) were stimulated with a range of imiquimod (TLR7) concentrations (50 pg·ml^−1^ to 100 µg·ml^−1^) to generate the response curves and resulted in group differences (P = 0.0048). Error bars on graphs represent standard error of the mean.

The TLR7 agonist imiquimod induced elevations in IL-1β release in only the CP+O group. The observed concentration-response curve was monophasic and due to the low amount of IL-1β expressed in response to TLR7 no response curve could represent the CP group and the controls. The differences were most prominent at the higher imiquimod concentrations causing a marked increase in the IL-1β (*F*_2,201_ = 5, *P* = 0.005; see Figure 3). The EC_50_ for CP+O was 545 µg·ml^−1^ (95% CI: 283 to 1048 µg·ml^−1^); CP, 2371 µg·ml^−1^ (95% CI: 1016 to 5535 µg·ml^−1^); and PF, 2172 µg·ml^−1^ (95% CI: 1345 to 3506 µg·ml^−1^) for PF.

#### (D) NALP3: TLR independent pathway: MSU crystals

The MSU crystals with one concentration of LPS (13 pg ml^−1^) induced expression of IL-1β in all three groups (data not shown). All groups responded with a similar expression of IL-1β at each of the tested concentrations that resulted in no significant group or concentration differences (group effect: *P* = 0.2 and concentration effect: *P* = 0.7). The range of IL-1β detected was similar to the amount released only by LPS stimulation. For example, at 1 µg ml^−1^ of MSU crystals with 13 pg ml^−1^ of LPS, the amount of IL-1β expressed in CP+O group was (mean ± SEM) 75±27 pg·ml^−1^ (LPS alone 13 pg ml^−1^ was 73±18 pg·ml^−1^), CP group was 112±34 pg·ml^−1^ (LPS alone 13 pg·ml^−1^ was 119±45 pg·ml^−1^) and by PF was 82±15 pg·ml^−1^ (LPS alone 13 pg·ml^−1^ was 35±18 pg·ml^−1^).

## Discussion

The current study, to the best of the authors' knowledge, is the first to examine the IL-1β expression responsiveness of isolated PBMCs collected from chronic pain sufferers (on and not on opioid treatments) following TLR agonist stimulation. Although basal levels of IL-1β did not differ between groups, after TLR agonist stimulation significant group differences were detected in the IL-1β expression levels. The response appears to be selective for TLR pathways as there was no response found in all 3 populations after MSU crystals stimulation, (an inflammasome activator). These findings supported both our hypotheses of increased TLR responsiveness in pain patients and further increases in TLR responsiveness in pain patients on opioids.

The general technique *of in vitro* stimulation of human PBMCs with TLR agonists followed by quantification of cytokine expression is not novel. This approach can be used to identify a patient's immunologic “signature” [26] and to understand the TLR signaling pathway. Studies to evaluate TLR responsiveness in chronic pain patients have been limited. Our study is the first to show enhanced TLR responsiveness in PBMCs collected from chronic pain patients when compared with controls. Previous studies have demonstrated enhanced pro-inflammatory cytokine release from TLR agonist stimulation in PBMCs from patients with rheumatoid arthritis [27], [28], primary biliary cirrhosis [29], inflammatory bowel diseases [30], chronic fatigue [26] and immunosuppressed patients with rheumatoid arthritis [31]. These conditions with the exception chronic fatigue, are defined by significant clinical inflammation and hence these findings are not surprising. We took care to exclude patients with clinical inflammation, suggesting that our findings are related to the pain state. The further enhanced sensitivity in patients taking opioids supports this. From the above studies, enhanced TLR-induced release of pro-inflammatory cytokines by PBMCs indicates a possible dysregulation in the innate immune system.

The increased responsiveness to TLR agonists could be attributable to the chronic pain patient's PBMC TLR system having been “primed” [32] following previous exposure of endogenous TLR ligands such as heat shock proteins [33] or HMGB-1 [34] which may be produced after a nerve injury. Such priming events are known to occur following painful insults. For example increased TLR2 mRNA [35], TLR4 mRNA and pro-inflammatory cytokine expression were found in murine models after L5 spinal nerve transection and sciatic nerve ligation [4], [36]. Interestingly, these changes in immune responsiveness are not limited to the CNS with a recent report of increased peripheral immune cell activity in a neuropathic pain model in mice [37]. It is therefore possible that the increased PBMC responsiveness observed in the chronic pain groups may be a result of up regulation of the expression of the TLR pathways. In addition, the different responsiveness between the TLR2 and TLR4 agonists could be attributed to the availability of the TLR4 accessory protein known as myeloid differentiation factor 2 (MD-2). LPS has to be captured by the LPS-binding protein then delivered to the TLR4/MD-2 complex that causes dimerization of the TLR4/MD-2 complex. In contrast, TLR2 does not need any accessory protein to activate downstream cascades [38]. Which pathways and the manner of alteration that lead to the changed responsiveness are a focus of future studies by our group.

Recently, a study unexpectedly found increased TLR4 surface expression on monocytes and enhanced pro-inflammatory cytokine (IL-8 and TNF-α) release after TLR stimulation (TLR1/2 heterodimer, TLR2/6 heterodimer, TLR4 and TLR5) in patients with autoimmune disease who were being treated with immunosuppressive medications, compared with patients not receiving immunosuppressive agents [31]. The authors hypothesized reduced TLR4 function as an explanation of increased infection associated with immunosuppressive therapy for autoimmune disease. The increased release of IL-8 and TNF-α after TLR stimulation was suggested to be as a result of altered TLR intracellular signaling because the surface expression of TLR1 and TLR2 did not differ from controls but the production of cytokines were enhanced. The innate immune system was concluded to be dysregulated and not simply suppressed. This finding parallels our study, despite differences in patient population, cell type and outcomes, where we observed enhanced immune response in patients receiving opioids despite opioid medications generally being regarded as being immunosuppressive [39] (see below).

Chronic morphine exposure is believed to cause immunosuppression as increased susceptibility to both bacterial and viral infections have been reported in drug users [39]. After morphine exposure, inhibition of the immune system, such as reduced monocyte function have been reported in humans [40] and decreased T-cell proliferation and NK cell function have been reported in rodents [41]. However the evidence is not all clearly in favor of immunosuppression, as hydrocodone and oxycodone were found to have no immunosuppressant properties with no effect on lymphocyte proliferation and natural killer activity found) [42]. Hence the concept of Dunne et al [31] of “dysregulation” may be more appropriate to the effects of opioids than simple concepts of enhanced or reduced immune activity. Such terms of enhanced or reduced immune activity fail to capture the complex interactions occurring within the immune system in which one part of the system may show increased activity while another part of action of the system is reduced.

A note of caution is needed with the assumption that the increased response in the CP+O group are causally related to the drugs using a cross sectional design. The CP+O group differed from the CP group by higher pain scores and duration of pain. It might seem paradoxical that patients on opioids have more pain, but since opioid use is recommended to be restricted to those with severe pain [43], [44], and as the drugs have only modest efficacy [43], [44] this finding is not surprising. Therefore, opioid use may be an indicator for more severe pain or pain of longer duration and these differences may be confounders in the interpretation. In future studies, controlling for pain intensity and duration between the groups might be interesting and would be important to examine in prospective longitudinal rather than cross-sectional study designs.

No group differences were found in the basal level of IL-1β release by PBMCs. This result is not surprising as basal level of cytokines have yielded inconsistent findings in other patient populations such as in rheumatoid arthritis patients. One study reported the basal level to be the same as controls [28] whilst another study found lower cytokine levels in rheumatoid arthritis patients compared to controls [27]. This could be due to the very low circulating cytokine levels but difference in sensitivity of the system may only be revealed after stimulation.

There is clear heterogeneity in inter-laboratory reporting of the sensitivity of PBMCs to LPS. At 1 ng/mL of LPS, the IL-1β released from PBMCs in the control group in our study (950 pg/mL) was higher than previous reports (600 pg/(5×10^5^/ml) [26]. Higher concentrations of LPS (1 µg/mL) produced lower amounts of IL-1β (2008 pg/mL) compared to other studies (5000 pg/mL) [45]. The differences could be attributed to the lack of a standard operating procedure and specific experimental conditions in the investigation of cytokine release from PBMCs. Other differences in the following will also influence the outcome: the serotype of TLR agonists used (e.g LPS), cell population used (mixed populations of cells versus monocytes only), method of PBMC isolation, cell concentration used and apparatus used for cytokine measurements (bead assay, multiplex ELISA and standard ELISA kits). No studies have investigated the TLR responsiveness of PBMCs from chronic pain patients. One key difference between our study and the bulk of the literature in the area is that we determined extensive concentration-response curves whereas in most other studies only a single concentration of agonist was used. Such single point determinations may miss the complexity of response, like the biphasic response we observed to LPS which has not been reported previously.

Biphasic cytokine responses (IL-8) have been reported following LPS stimulation in whole blood [46]. However, in this study the secondary wave observed was due to the effect of time (first wave was from 6–12 hours and the secondary wave was from 12–24 hours) instead of concentration of LPS (as was observed in the current study). The secondary wave was attributed to the LPS-induced release of TNF and IL-1 as demonstrated with the disappearance of the wave with the use of anti-TNF and anti-IL-1 neutralizing antibodies. In the current study the biphasic nature of the IL-1 response may have arisen from the activation of two systems (possibly in two cell types) that are characterized as high affinity but low capacity, verses lower affinity but higher capacity. This response characteristic may not have been examined before as most studies usually only employ one high concentration of agonist.

The specificity of the TLR agonists response was tested with the use of the non-TLR signaling stimulus, monosodium urate monohydrate (MSU) crystals [47]. MSU crystals are reported to be an inflammasome activator and cause the conversions of procaspase-1 to active caspase 1 allowing pro-IL-1β can be cleaved to active IL-1β via the NALP3 inflammasome [48]. No detectable IL-1β from collected PBMCs were found with pure MSU crystals. Therefore, a low concentration of LPS (second stimulus) was added with MSU crystal so the synergistic activation could be measured. However, no group differences were detected between the 3 groups. Hence it confirms our finding of the increased responsiveness in pain patients may be TLR-specific and not related to other pathway such as the NALP3 inflammasome.

Previously, our group found a strong correlation (Pearson r  = 0.9) between morphine-enhanced concanavalin A proliferation (in isolated PBMCs) with cold pain endurance score in a healthy voluntary population[49]. Therefore we wished to investigate whether cold pain endurance could be correlated with TLR agonist induced IL-1β levels in the PBMCs collected in the current experiment. However no group differences were found with the cold pain endurance scores and no correlation was found with the TLR agonists.

Some limitations of this study are: (1) the small sample size and heterogeneity of pain diagnosis and severity. The heterogeneity was deliberate as we hypothesized that it was the chronic pain process *per se* and not the underlying diagnosis that was associated with increased immune responsiveness. In future studies of larger sample size, selecting a range of clearly defined pathologies and controlling for pain severity would be useful. (2) Potential confounding effects of concomitant depression and changes in the in the hypothalamic-pituitary-adrenal axis status were not studied, but in future work this would be appropriate as depression may be associated with changes in glial activity [50]. (3) Only one pro-inflammatory cytokine was measured and IL-1β was chosen as it plays a pivotal role in chronic pain. Future studies should examine a broader range of cytokines and chemokines.

In summary, TLR agonists (TLR2, TLR4, and TLR7) were found to cause elevation in IL-1β release that could significantly differentiate from chronic pain sufferers on opioids, chronic pain sufferers not on opioids and pain-free participants. This study is the first in providing evidence in human cells that TLRs are more responsive in chronic pain sufferers. As we were able to significantly differentiate three groups on the basis of their IL-1β output, it appears this response of *in vitro* stimulation of isolated immune cells with TLR agonists may serve to be a potential test for identifying biomarkers for chronic pain from readily accessible peripheral blood samples.

## Supporting Information

Table S1 **Individual information on chronic pain sufferers in this study.**
(DOCX)
