# Supplementary material for: Increased Responsiveness of Peripheral Blood Mononuclear Cells to In Vitro TLR 2, 4 and 7 Ligand Stimulation in Chronic Pain Patients
Source: PLoS One. 2012 Aug 28;7(8):e44232. doi: 10.1371/journal.pone.0044232 (PMC3429430; doi:10.1371/journal.pone.0044232)
Supplement: Table S1 — Individual information on chronic pain sufferers in this study. (DOCX) [file pone.0044232.s001.docx]

**Table S1.**

| **Participant** | **Age/ Gender** | **Primary pain diagnosis** | **Opioid** | **Other analgesics** | **oral morphine equivalent dose (mg)** | **Duration of pain (years)** |
| --- | --- | --- | --- | --- | --- | --- |
| **Group 1: Chronic Pain sufferers on opioid** | | | | | |  |
| 1 | 63/F | Sciatica | Morphine | Amitriptyline, pregabalin and paracetamol | 200 | 10 |
| 2 | 43/M | Chronic back and leg pain | Oxycodone |  | 80 | 28 |
| 3 | 65/F | Osteoarthritis | Oxycodone |  | 120 | 3 |
| 4 | 49/F | Chronic low back pain | Oxycodone | Diclofenac | 36 | 16 |
| 5 | 54/M | Osteoarthritis of hip | Oxycodone |  | 80 | 2 |
| 6 | 59/F | Back and shoulder pain | Methadone |  | 30 | 20 |
| 7 | 45/M | Osteoarthritis | Morphine | Venlafaxine, quetiapine and diazepam | 70 | 3 |
| 8 | 42/M | Fibromyalgia | Paracetamol/ codeine | Duloxetine | 30 | 3 |
| 9 | 64/F | Osteoarthritis | Tramadol | Celecoxib and paracetamol/ codeine | 107 | 14 |
| 10 | 62/F | Back Pain & migraine | Morphine | Oxycodone hydrochloride and paracetamol/ codeine | 40 | 7 |
| 11 | 33/F | Complex regional pain syndrome | Oxycodone | Pregabalin and paracetamol/ codeine | 120 | 7 |
| **Group 2: Chronic pain sufferers not on opioids** | | | | | |  |
| 12 | 36/F | Osteoarthritis of knee | None | Ibuprofen | N/A | 2 |
| 13 | 65/M | Osteoarthritis of knee | None | Ibuprofen# | N/A | 1 |
| 14 | 60/M | Sciatica | None | Citalopram and paracetamol# | N/A | 4 |
| 15 | 40/F | Atypical trigeminal neuralgia | None | Amitriptyline#, carbamazepine# and pregabalin# | N/A | 2 |
| 16 | 64/F | Chronic low back pain | None |  | N/A | 11 |
| 17 | 45/F | Neuropathic pain syndrome | None | Gabapentin, amitriptyline | N/A | 4 |
| 18 | 57/F | Non cardiac chest pain | None | Paracetamol# | N/A | 4 |
| 19 | 46/F | Fibromyalgia and back pain | None |  | N/A | 5 |

# denotes when required
